# Association between *CTLA-4* gene polymorphism and risk of rheumatoid arthritis: a meta-analysis

**DOI:** 10.18632/aging.203349

**Published:** 2021-08-02

**Authors:** Chuankun Zhou, Shutao Gao, Xi Yuan, Zixing Shu, Song Li, Xuying Sun, Jun Xiao, Hui Liu

**Affiliations:** 1 Department of Orthopedics, Tongji Hospital, Tongji Medical College, Huazhong University of Science and Technology, Wuhan 430030, Hubei, China; 2 Department of Spine Surgery, The First Affiliate Hospital of Xinjiang Medical University, Urumqi 830054, Xinjiang, China; 3 Department of Orthopedics Trauma and Microsurgery, Zhongnan Hospital, Wuhan University, Wuhan 430000, Hubei, China

**Keywords:** CTLA-4, polymorphism, rheumatoid arthritis, meta-analysis

## Abstract

Cytotoxic T lymphocyte-associated protein 4 (*CTLA-4*) gene polymorphisms may be involved in the risk of Rheumatoid arthritis (RA). However, evidence for the association remains controversial. Therefore, we performed a meta-analysis to confirm the relationship between *CTLA-4* gene polymorphisms and RA. The pooled odds ratios (ORs) and 95% confidence intervals (CIs) were calculated to assess the strength of association. Stratified analysis was conducted by ethnicity. In total, 66 case-control studies including 21681 cases and 23457 controls were obtained. For rs3087243 polymorphism, significant association was detected in Asians (*A* vs. *G*: OR=0.77, 95%CI=0.65-0.90, *P*=0.001; *AA* vs. *GG*: OR=0.67, 95%CI=0.48-0.94, *P*=0.02) and Caucasians (*A* vs. *G*: OR=0.89, 95%CI=0.86-0.93, *P*<0.00001; *AA* vs. *GG*: OR=0.81, 95%CI=0.75-0.88, *P*<0.00001). For rs231775 polymorphism, significant association was observed in the overall (*G* vs. *A*: OR =1.16, 95%CI=1.08-1.25, *P*<0.0001; *GG* vs. *AA*: OR=1.29, 95%CI=1.12-1.50, *P*=0.0006), and in Asians (*G* vs. *A*: OR=1.27, 95%CI=1.10-1.47, *P*=0.001; *GG* vs. *AA*: OR=1.58, 95%CI=1.24-2.01, *P*=0.0002), but not in Caucasians. However, there was no association between rs5742909 polymorphism and RA. This meta-analysis confirmed that rs3087243 and rs231775 polymorphism were associated with the risk of RA in both overall population and ethnic-specific analysis, but there was no association between rs5742909 polymorphism and RA risk.

## INTRODUCTION

Rheumatoid arthritis, one of the most common inflammatory joint diseases in humans, is characterized by inflammation in synovium, destruction of cartilage and bone, generation of autoantibody, and complications of systemic organs [1]. Although RA affects 0.5–1% of the Western populations, the worldwide incidence of RA is increasing with the aging trend of the population [2]. Because of the results of reduced physical function, declined work capacity, decreased quality of life, and increased comorbid risk, RA carries heavy socioeconomic burden [3]. RA is believed to be a consequence of both genetic factors and environmental factors though main etiology has not yet been clearly clarified. In twin studies 50–65% of the risk for developing RA is ascribed to its heritability [4], indicating genetic factors have a strong effect on RA. So far more than one hundred gene loci associated with RA risk have been identified by single nucleotide polymorphisms (SNPs) [5, 6]. Apart from the human leukocyte antigen (HLA) locus, a well-known genetic risk factor for RA, numbers of other susceptibility genes and loci have been characterized [6]. Recently, a growing body of non-HLA genetic predisposition studies have been conducted on the association with the risk of RA [7–9].

Cytotoxic T lymphocyte-associated protein 4 (*CTLA-4*), one of widely studied non-HLA susceptibility gene of RA, is mainly expressed on the surface of Treg cells and conventional T cells and suppresses self-reactive T cell responses via downregulating ligand availability for the costimulatory receptor CD28 to elicit inhibitory signals [10, 11]. Besides, the polymorphisms of *CTLA-4* have already been proved to be candidates of the risk of the common autoimmune diseases at the genetic level [12–15]. As RA is a T cell mediated autoimmune disorder and *CTLA-4* plays a vital role in regulating T cell function [11, 12, 16], it suggests that *CTLA-4* expression or function is most likely associated with the pathogenesis of RA. Single nucleotide polymorphisms in the *CTLA-4* gene may contribute to abnormal levels of *CTLA-4*, and subsequently play a leading part in the susceptibility to RA [12, 17, 18].

Among the identified SNPs in this gene, these three loci of *CTLA-4*, +49*A/G* (rs231775), -318*C/T* (rs5742909) and CT60 *G/A*(rs3087243), are most-often studied for the association with the predisposition of RA [18–20]. However, the conclusions which previous reports drew are inconsistent and incomprehensive. Although the association of *CTLA-4* genetic polymorphisms and the risk of RA has been assessed in several meta-analyses [21–23], some recent studies also described this association in different populations in the past several years [9, 15, 24–27]. Hence these studies should be included to increase statistical power and obtain the reliable conclusion. On the other hand, all the three common loci should be included to embody the association comprehensively while the previous meta-analysis only researched one or two of the above loci. In view of these, it is necessary to incorporate the latest research into investigating the association of the three polymorphisms of *CTLA-4* with susceptibility to RA. Here we use the latest case-control data to carry out an updated and comprehensive meta-analysis and obtain a more accurate estimation of the effect of the 3 SNPs (+49*A/G* (rs231775), CT60 *G/A*(rs3087243) and -318 *C/T* (rs5742909)) on RA risk.

## RESULTS

### Characteristics of the studies

**Figure 1:**
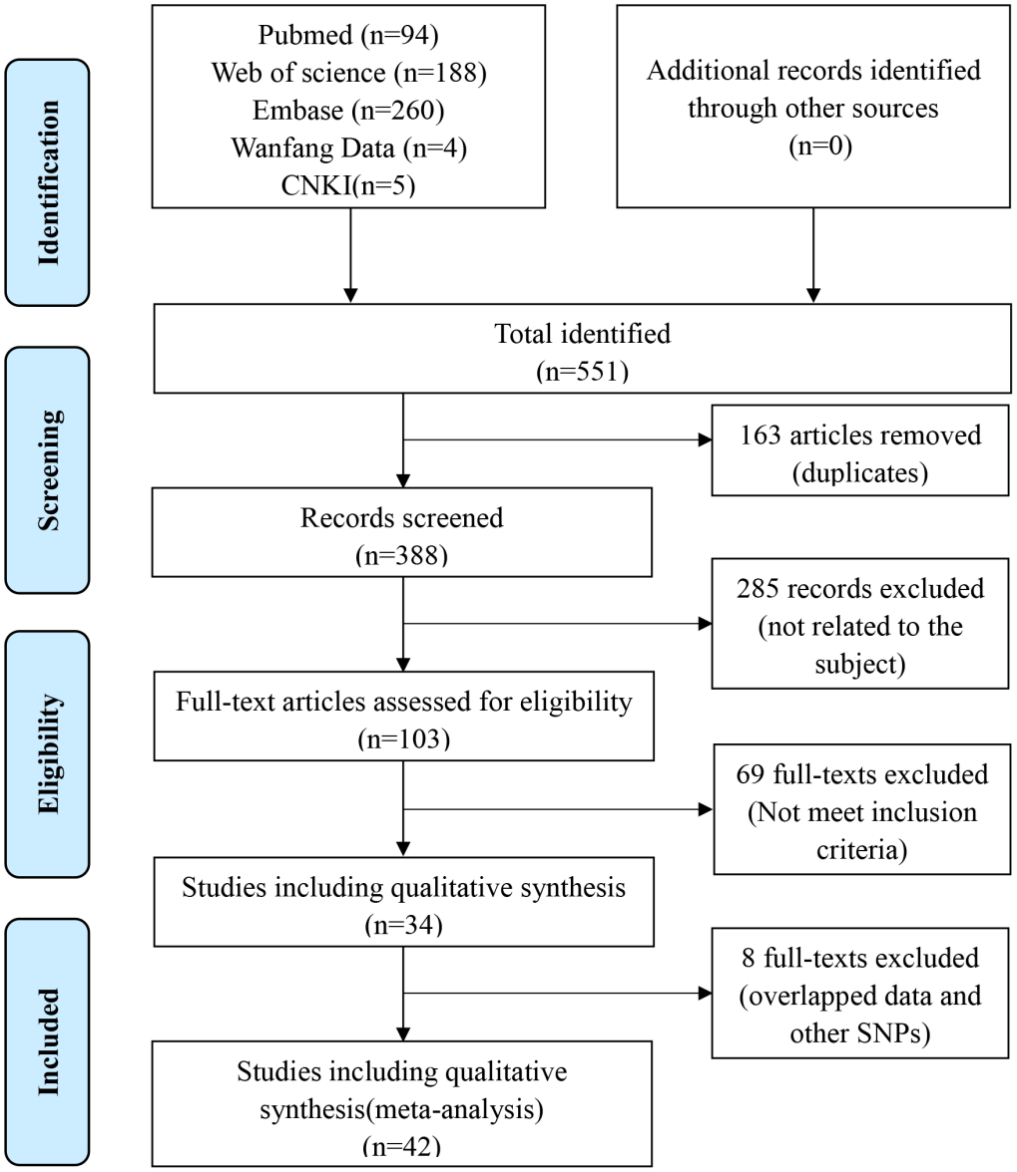
Flow diagram of the literature retrieval and screen.

Based on the predetermined inclusion criteria, 66 eligible case–control studies with 42 articles were enrolled ultimately in the current analysis [8, 9, 13–15, 17–20, 24–56]. These publications had a high methodological quality whose NOS stars were more than 6 in general. There were 22 studies with 16394 patients and 17453 controls for rs3087243 SNP [8, 9, 13–15, 18, 19, 26, 40, 41, 43, 46–49, 52, 53, 56], 34 studies with 11452 patients and 12444 controls for rs231775 SNP [9, 14, 17, 19, 20, 24, 25, 28–39, 41–45, 49–51, 54], and 10 studies with 2477 patients and 2941 controls for rs5742909 SNP [14, 20, 27, 29, 34, 37–39, 44, 56]. The references of all enrolled articles were subject to scrutiny and no more ones were available. The process of study selection according to the PRISMA principle was generalized in Figure 1. Quality assessment of included studies was shown in Supplementary Table 1. Details of included studies were listed in Table 1. Allele/genotype frequencies were displayed in Table 2.

**Table 1:** Main characteristics of included studies.

**Study**	**Year**	**Country**	**Ethnicity**	**Numbers**		**Genotype** **method**	**Diagnostic criteria**	**Quality** **score**
				**RA**	**Con**			
**Rs3087243(CT60)**								
Orozco	2004	Spain	Caucasian	433	398	TaqMan	ACR1987	7
Lei	2005	China	Asian	326	250	DGGE	ACR1987	8
Plenge (EIRA)	2005	Sweden	European	1505	878	MALDI-TOF	ACR1987	8
Plenge (NARAC)	2005	Sweden	European	828	845	MALDI-TOF	ACR1987	8
Zhernakova	2005	Dutch	Caucasian	153	900	PCR-RFLP	ACR1987	6
Suppiah	2006	Northern Ireland	Caucasian	289	168	PCR-RFLP	ACR1987	7
Costenbader	2008	USA	Caucasian	423	420	TaqMan	ACR1987	7
Tsukahara	2008	Japan	Asian	1498	441	TaqMan	ACR1987	8
Kelley	2009	USA	African	505	712	TaqMan	ACR1987	7
Daha	2009	Dutch	Caucasian	867	863	Sequenom	ACR1987	7
Barton	2009	UK	European	3669	3049	TaqMan	ACR1987	8
Walker	2009	Canada	Caucasian	1140	1248	Sequenom	ACR1987	8
Plant (1)	2010	France	Caucasian	671	177	Sequenom	ACR1987	8
Plant (2)	2010	Germany	Caucasian	218	209	Sequenom	ACR1987	8
Plant (3)	2010	Greece	Caucasian	268	290	Sequenom	ACR1987	8
Plant (4)	2010	UK	Caucasian	1002	2725	Sequenom	ACR1987	8
Danoy	2011	China	Asian	1035	1702	Sequenom	ACR1987	7
Torres-Carrillo	2013	Mexico	Latin American	200	200	PCR–RFLP	ACR1987	8
Luterek-Puszyńska	2016	Poland	Caucasian	422	338	TaqMan	ACR1987	7
Schulz	2020	Germany	Caucasian	111	256	PCR–RFLP	ACR2010	6
El-Gabalawy	2011	Canada	Caucasian	332	490	Sequenom	ACR1987	6
Vernerova	2016	Slovakia	Caucasian	499	894	TaqMan	ACR2010	9
**Rs231775(49G/A)**								
AIFadhli	2013	Kuwait	Asian	114	282	PCR–RFLP	ACR1987	6
Barton (I)	2000	Spain	Caucasian	136	144	PCR–RFLP	ACR1987	7
Barton (II)	2000	UK	Caucasian	192	96	PCR–RFLP	ACR1987	7
Benhatchi	2011	Slovakia	Caucasian	57	51	PCR–RFLP	ACR1987	6
Elshazli	2015	Egypt	Caucasian	112	122	PCR–RFLP	ACR1987	6
Feng	2005	China	Asian	50	60	PCR–RFLP	ACR1987	6
Gonzalez-Escribano	1999	Spain	Caucasian	138	305	PCR-ARMS	ACR1987	6
Hadj	2001	Tunisia	African	60	150	PCR–RFLP	ACR1987	7
Lee 2002	2002	Korea	Asian	86	86	PCR–RFLP	ACR1987	6
Lee 2003	2003	China	Asian	186	203	PCR–RFLP	ACR1987	6
Lei	2005	China	Asian	326	250	DGGE	ACR1987	8
Liu 2004	2004	Taiwan	Asian	65	81	PCR–RFLP	ACR1987	6
Barton	2004	UK	European	132	156	TaqMan	ACR1987	7
Liu 2013	2013	China	Asian	213	303	PCR–RFLP	ACR1987	7
Luterek-Puszyńska	2016	Poland	Caucasian	422	338	TaqMan	ACR2010	7
Matsushita	1999	Japan	Asian	461	150	PCR-SSCP	ACR1987	7
Milicic	2001	UK	Caucasian	421	452	PCR–RFLP	ACR1987	8
Miterski	2004	Germany	Caucasian	284	362	PCR–RFLP	ACR1987	7
Munoz-Valle	2010	Mexico	Mexican	199	199	PCR–RFLP	ACR1987	6
Plant (1)	2010	France	Caucasian	684	162	Sequenom	ACR1987	8
Plant (2)	2010	Germany	European	220	209	Sequenom	ACR1987	8
Plant (3)	2010	Greece	European	272	287	Sequenom	ACR1987	8
Plant (4)	2010	UK	European	1004	2659	Sequenom	ACR1987	6
Seidl	1998	Germany	Caucasian	258	456	RFLP-SSCP	ACR1987	8
Suppiah	2006	UK	European	289	475	PCR–RFLP	ACR1987	7
Takeuchi	2006	Japan	Asian	100	104	PCR–RFLP	ACR1987	6
Tang	2013	China	Asian	1489	1200	TaqMan	ACR1987	8
Tsukahara	2008	Japan	Asian	1490	448	TaqMan	ACR1987	8
Kelley	2009	USA	African	505	712	TaqMan	ACR1987	7
Vaidya	2002	UK	Caucasian	123	349	PCR–RFLP	ACR1987	6
Walker	2009	Canada	Caucasian	1140	1248	Sequenom	ACR1987	8
Yanagawa	2000	Japan	Asian	85	200	PCR–RFLP	ACR1987	6
Zhou	2007	China	Asian	39	44	PCR–RFLP	ACR1987	6
Sameem	2015	Pakistani	Asian	100	100	PCR–RFLP	RF test	6
**Rs5742909 (318C/T)**								
Gonzalez-Escribano	1999	Spain	Caucasian	138	305	PCR-ARMS	ACR1987	6
Lee 2002	2002	Korea	Asian	86	86	PCR–RFLP	ACR1987	6
Barton	2004	UK	European	151	152	TaqMan	ACR1987	7
Liu 2004	2004	Tainan	Asian	65	81	PCR–RFLP	ACR1987	6
Miterski	2004	Germany	Caucasian	284	362	PCR–RFLP	ACR1987	7
Takeuchi	2006	Japan	Asian	100	104	PCR–RFLP	ACR1987	6
Walker	2009	Canada	Caucasian	1140	1248	Sequenom	ACR1987	8
Liu 2013	2013	China	Asian	213	303	PCR–RFLP	ACR1987	7
Torres-Carrillo	2013	Mexico	Latin American	200	200	PCR–RFLP	ACR1987	7
Fattah	2017	Egypt	Caucasian	100	100	PCR–RFLP	ACR2010	6

**Table 2:** Distribution of genotype and allele among RA patients and controls.

**Study**	**Cases**						**Controls**					**HEW**
	**MM**	**Mm**	**mm**	**M**	**m**		**MM**	**Mm**	**mm**	**M**	**m**	
**Rs3087243(CT60)**												
Orozco	118	198	117	434	432		98	199	101	395	401	YES
Lei	33	137	156	203	449		32	131	87	195	305	YES
Plenge (EIRA)	230	680	595	1140	1870		145	396	337	686	1070	YES
Plenge (NARAC)	133	387	308	653	1003		165	426	254	756	934	YES
Zhernakova	NA	NA	NA	133	173		NA	NA	NA	841	959	NA
Suppiah	NA	NA	NA	234	344		NA	NA	NA	145	191	NA
Costenbader	82	201	140	365	481		87	195	138	369	471	YES
Tsukahara	87	538	873	712	2284		33	163	245	229	653	YES
Kelley	NA	NA	NA	NA	505		NA	NA	NA	NA	712	NA
Daha	NA	NA	NA	729	1005		NA	NA	NA	785	941	NA
Barton	677	1760	1232	3114	4224		634	1523	892	2791	3307	YES
Walker	207	518	415	932	1348		273	613	362	1159	1337	YES
Plant (1)	131	332	208	594	748		45	91	41	181	173	YES
Plant (2)	35	105	78	175	261		35	101	73	171	247	YES
Plant (3)	55	135	78	245	291		70	145	75	285	295	YES
Plant (4)	204	487	311	895	1109		542	1344	839	2428	3022	YES
Danoy	NA	NA	NA	310	1760		NA	NA	NA	681	2723	NA
Torres-Carrillo	31	86	83	148	252		32	106	62	170	230	YES
Luterek-Puszyńska	53	193	176	299	545		45	174	119	264	412	YES
Schulz	13	49	49	75	147		42	124	90	208	304	YES
El-Gabalawy	45	161	126	251	413		66	226	198	358	622	YES
Vernerova	NA	NA	NA	616	382		NA	NA	NA	1064	1064	NA
**Rs231775(49G/A)**												
AIFadhli	10	30	74	50	178		14	86	182	114	450	YES
Barton (I)	14	57	65	85	187		12	70	62	94	194	YES
Barton (II)	38	86	68	162	222		19	51	26	89	103	YES
Benhatchi	6	33	18	45	69		5	25	21	35	67	YES
Elshazli	14	55	43	83	141		6	45	71	57	187	YES
Feng	20	21	9	61	39		9	32	19	50	70	YES
Gonzalez-Escribano	10	63	65	83	193		30	103	172	163	447	NO
Hadj	23	27	10	73	47		68	62	20	198	102	YES
Lee 2002	41	35	10	117	55		49	29	8	127	45	YES
Lee 2003	103	67	16	273	99		85	100	18	270	136	YES
Lei	148	138	40	434	218		86	125	39	297	203	YES
Liu 2004	14	42	9	70	60		21	50	10	92	70	NO
Barton	34	55	43	123	141		29	68	59	126	186	YES
Liu 2013	77	111	25	265	161		130	125	48	385	221	YES
Luterek-Puszyńska	79	210	133	368	476		63	160	115	286	390	YES
Matsushita	200	199	62	599	323		56	72	22	184	116	YES
Milicic	63	223	135	349	493		73	213	166	359	545	YES
Miterski	NA	NA	NA	222	346		NA	NA	NA	269	455	NA
Munoz-Valle	42	102	55	186	212		34	82	83	150	248	YES
Plant (1)	96	315	273	507	861		15	75	72	105	219	YES
Plant (2)	37	111	72	185	255		32	94	83	158	260	YES
Plant (3)	26	133	113	185	359		33	107	147	173	401	YES
Plant (4)	146	451	407	743	1265		410	1255	994	2075	3243	YES
Seidl	37	138	83	212	304		68	210	179	346	568	YES
Suppiah	40	144	105	224	354		92	241	142	425	525	YES
Takeuchi	49	39	12	137	63		44	49	11	137	71	YES
Tang	652	642	195	1946	1032		474	535	191	1483	917	YES
Tsukahara	636	668	186	1940	1040		181	194	73	556	340	YES
Kelley	NA	NA	NA	NA	505		NA	NA	NA	NA	712	NA
Vaidya	20	65	38	105	141		45	158	146	248	450	YES
Walker	177	554	409	908	1372		178	577	493	933	1563	YES
Yanagawa	29	50	6	108	62		78	88	34	244	156	YES
Zhou	22	9	8	53	25		8	14	22	30	58	YES
Sameem	54	26	20	134	66		28	31	41	87	113	NO
**Rs5742909 (318C/T)**												
Gonzalez-Escribano	1	29	108	31	245		2	60	243	64	546	NO
Lee 2002	2	19	65	23	149		4	14	68	22	150	YES
Barton	1	18	132	20	282		3	27	122	33	271	YES
Liu 2004	0	15	50	15	115		0	23	58	23	139	NO
Miterski	NA	NA	NA	64	504		NA	NA	NA	50	674	NA
Takeuchi	0	13	87	13	187		0	22	82	22	186	YES
Walker	13	219	908	245	2035		10	183	1055	203	2293	YES
Liu 2013	14	97	102	125	301		13	77	213	103	503	YES
Torres-Carrillo	2	16	182	20	380		0	20	180	20	380	YES
Fattah	7	52	41	66	134		2	32	66	36	164	YES

M, minor allele; m, major allele; NA, not available; HWE, Hardy-Weinberg Equilibrium.

### Efficiency analysis

### Meta-analysis of CTLA-4 CT60(rs3087243) SNP and RA susceptibility

**Figure 2:**
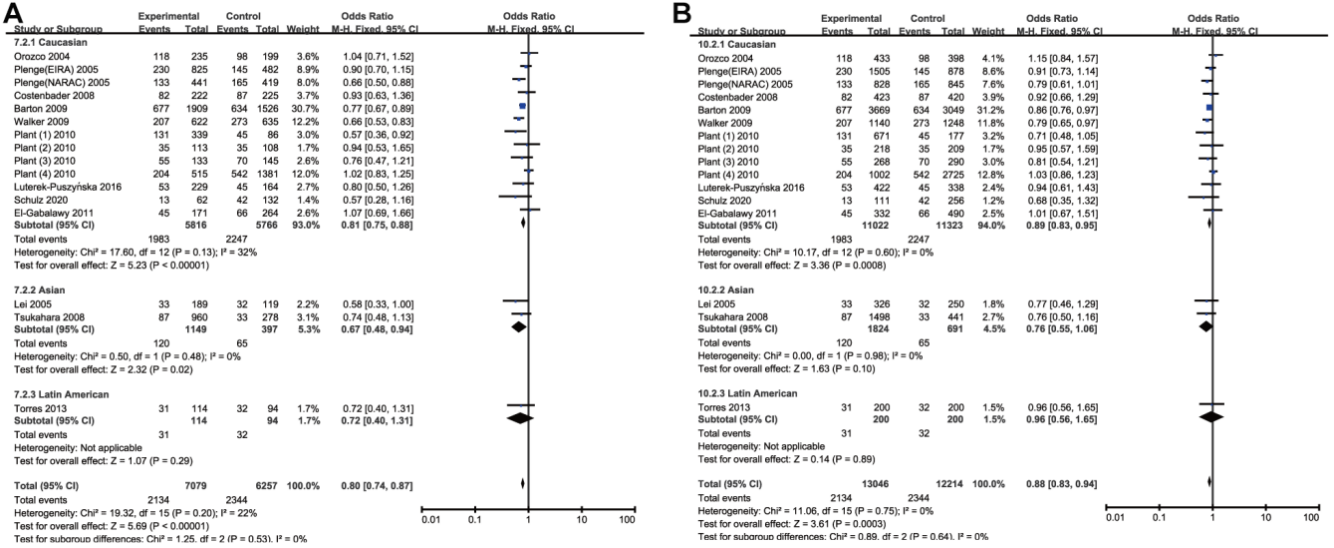
Forest plot of the association between rs308724 polymorphism and RA risk under the homozygous (**A**) and recessive model (**B**).

By analyzing quantitatively allele or genotype distribution of 16394 patients and 17453 controls, a significant association between RA and *CTLA-4* CT60(rs3087243) SNP was observed in all genetic comparisons (*A* vs. *G*: OR = 0.87, 95% CI = 0.83-0.91, *P*<0.00001; *AA* vs. *GG*: OR = 0.80, 95% CI =0.74-0.87, *P*<0.00001; *AG* vs. *AA*: OR = 0.85, 95% CI =0.80-0.90, *P*<0.0001; *AA* + *AG* vs. *GG*: OR =0.83, 95% CI=0.77-0.90, *P*<0.0001, and *AA* vs. *AG*+ *GG*: OR =0.88, 95% CI=0.83-0.94, *P*=0.0003) (Table 3 and Figure 2). Among the 22 included studies, 17 studies were performed in Caucasians, 3 were in Asians, 1 was African and 1 was in Latin Americans. Likewise, we carried out a stratified analysis by race to evaluate the ethnicity effects. In Caucasians, a protective role of rs3087243 SNP on RA was detected in all the five genetic comparisons. Similarly, a decreased risk of RA was found among Asians in the allelic comparison (OR = 0.77, 95% CI =0.65-0.90, *P*=0.001) and the homozygote comparison (OR = 0.67, 95% CI = 0.48-0.94, *P*=0.02). The heterozygote model and dominant model detected also this correlation in Latin Americans and the allelic comparison detected this correlation in Africans, but both the two populations needed more enrolled studies to elevate statistical power because this analysis currently included individually only one study. The outcomes were shown in Table 3. Collectively, Subgroup analyses revealed a significant protective association in Caucasians and Asians. When the *I*^2^ > 50% and *P*>0.1, the Fix-effect model was used for the synthesis; otherwise, the Random-effect model was used.

**Table 3:** Results of different comparative genetic models on the association of CTLA-4 SNPs with RA.

**Genetic model**	**Population**	**Cases**	**Controls**		**Association**				**Heterogeneity**		
					**OR**	**95%CI**	***P-*value**		**Model**	***I*^2^**	***P-*value**
**Rs308724**											
A vs. G	Total	16394	17453		0.87	0.83-0.91	<0.00001		REM	39	0.003
	Caucasian	12830	14148		0.89	0.86-0.93	<0.00001		FEM	25	0.17
	Asian	2859	2393		0.77	0.65-0.90	0.001		REM	56	0.10
	Latin	200	200		0.79	0.60-1.06	0.11		¯	¯	¯
	African	505	712		0.83	0.67-1.02	0.08		¯	¯	¯
AA vs. GG	Total	13046	12214		0.80	0.74-0.87	<0.00001		FEM	22	0.20
	Caucasian	11022	11323		0.81	0.75-0.88	<0.00001		FEM	32	0.13
	Asian	1824	691		0.67	0.48-0.94	0.02		FEM	0	0.48
	Latin	200	200		0.72	0.40-1.31	0.29		¯	¯	¯
AG vs. GG	Total	13046	12214		0.85	0.80-0.90	<0.0001		FEM	28	0.14
	Caucasian	11022	11323		0.86	0.81-0.92	<0.0001		FEM	11	0.33
	Asian	1824	691		0.75	0.48-1.18	0.21		REM	78	0.03
	Latin	200	200		0.61	0.39-0.94	0.02		¯	¯	¯
AA+GA vs. GG	Total	13046	12214		0.83	0.77-0.90	<0.0001		REM	46	0.02
	Caucasian	11022	11323		0.85	0.78-0.93	<0.0002		REM	40	0.07
	Asian	1824	691		0.74	0.48-1.12	0.15		REM	77	0.04
	Latin	200	200		0.60	0.40-0.90	0.01		¯	¯	¯
AA vs. GA+GG	Total	13046	12214		0.88	0.83-0.94	0.0003		FEM	0	0.75
	Caucasian	11022	11323		0.89	0.83-0.95	0.0008		FEM	0	0.60
	Asian	1824	691		0.76	0.55-1.06	0.10		FEM	0	0.98
	Latin	200	200		0.96	0.56-1.65	0.89		¯	¯	¯
**Rs231775**											
G vs. A	Total	11452	12444		1.16	1.08-1.25	<0.0001		REM	66	0.00001
	Caucasian	5884	7872		1.09	1.01-1.19	0.04		REM	38	0.004
	Asian	4804	3511		1.27	1.10-1.47	0.001		REM	71	<0.0001
	African	565	862		1.06	0.68-1.65	0.81		REM	73	0.05
	Latin	199	199		1.45	1.09-1.92	0.010		¯	¯	¯
GG vs. AA	Total	10663	11370		1.29	1.12-1.50	0.0006		REM	54	0.0002
	Caucasian	5600	7510		1.11	0.94-1.31	0.21		FEM	25	0.17
	Asian	4804	3511		1.58	1.24-2.01	0.0002		REM	51	0.01
	African	60	150		0.68	0.28-1.65	0.39		¯	¯	¯
	Latin	199	199		1.24	1.09-1.42	0.03		¯	¯	¯
GA vs. AA	Total	10663	11370		1.19	1.07-1.32	0.001		REM	46	0.003
	Caucasian	5600	7510		1.18	1.02-1.35	0.02		REM	59	0.001
	Asian	4804	3511		1.20	1.05-1.38	0.08		FEM	3	0.42
	African	60	150		0.87	0.36-2.11	0.76		¯	¯	¯
	Latin	199	199		1.88	1.20-2.94	0.006		¯	¯	¯
GG+GA vs. AA	Total	10663	11370		1.24	1.11-1.39	0.0001		FEM	56	0.001
	Caucasian	5600	7510		1.17	1.02-1.34	0.02		REM	62	0.0006
	Asian	4804	3511		1.33	1.17-1.51	<0.0001		FEM	31	0.12
	African	60	150		0.77	0.34-1.76	0.53		¯	¯	¯
	Latin	199	199		1.87	1.23-2.85	0.003		¯	¯	¯
GG vs. GA+AA	Total	10663	11370		1.15	1.02-1.30	0.02		REM	57	<0.0001
	Caucasian	5600	7510		1.01	0.91-1.12	0.80		FEM	10	0.34
	Asian	4804	3511		1.34	1.08-1.65	0.008		REM	72	<0.0001
	African	60	150		0.75	0.41-1.38	0.36		¯	¯	¯
	Latin	199	199		1.30	0.79-2.15	0.31		¯	¯	¯
**Rs5742909**											
T vs. C	Total	2477	2941		1.21	0.93-1.57	0.15		REM	71	0.0003
	Caucasian	1813	2167		1.31	0.94-1.84	0.11		REM	73	0.005
	Asian	464	574		1.05	0.56-1.96	0.88		REM	80	0.002
	Latin	200	200		1.00	0.53-1.89	1.00		¯	¯	¯
TT vs. CC	Total	2193	2579		1.71	1.08-2.73	0.08		FEM	17	0.30
	Caucasian	1529	1805		1.58	0.60-4.17	0.35		REM	32	0.22
	Asian	464	574		1.34	0.34-5.28	0.68		REM	56	0.13
	Latin	200	200		4.95	0.24-103.73	0.30		¯	¯	¯
TC vs. CC	Total	2193	2579		1.19	0.84-1.69	0.33		FEM	76	<0.0001
	Caucasian	1529	1805		1.27	0.81-1.99	0.29		REM	74	0.01
	Asian	464	574		1.16	0.53-2.56	0.70		REM	83	0.0004
	Latin	200	200		0.79	0.40-1.58	0.51		¯	¯	¯
TT+TC vs. CC	Total	2193	2579		1.19	0.84-1.69	0.33		FEM	77	<0.0001
	Caucasian	1529	1805		1.28	0.79-2.07	0.32		REM	78	0.003
	Asian	464	574		1.12	0.52-2.43	0.77		REM	84	0.0003
	Latin	200	200		0.89	0.46-1.74	0.73		¯	¯	¯
TT vs. TC+CC	Total	2193	2579		1.43	0.90-2.27	0.13		FEM	0	0.52
	Caucasian	1529	1805		1.46	0.77-2.78	0.25		FEM	0	0.39
	Asian	464	574		1.27	0.63-2.54	0.51		FEM	32	0.23
	Latin	200	200		5.05	0.24-105.86	0.30		¯	¯	¯

OR, odds ratio; CI, confidence interval; FEM, fix-effect model; REM, random-effect model.

### Meta-analysis of CTLA-4 +49A/G (rs231775) SNP and RA susceptibility

**Figure 3:**
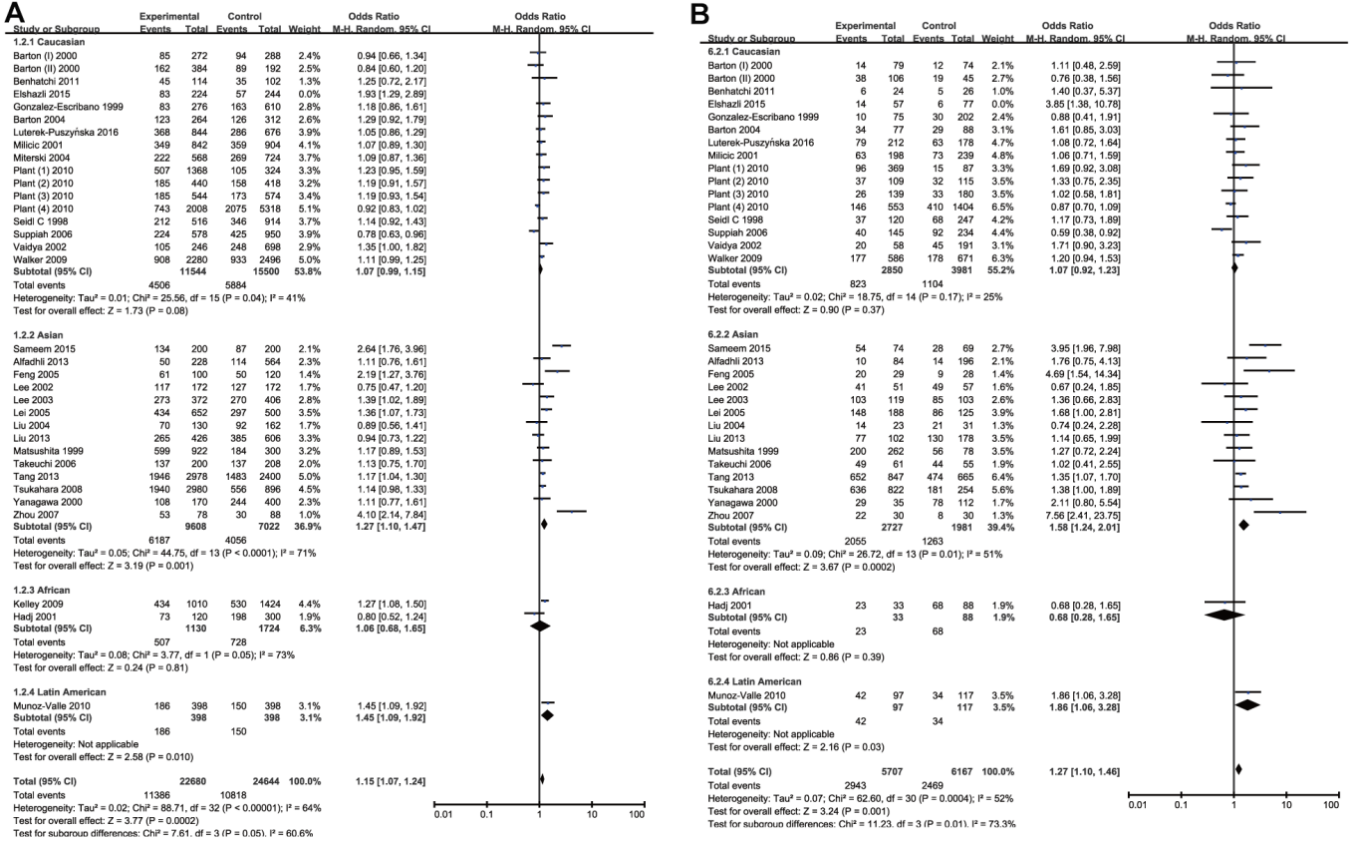
Forest plot of the association between rs231775 polymorphism and RA risk under the allelic model with Elshazli R et al.’s study excluded (**A**) and homozygous model (**B**).

By quantitative analysis of allele or genotype distribution of 11452 patients and 12444 controls, there was a significant risk association between RA and *CTLA-4* +49*A/G* (rs231775) SNP. The overall pooled ORs of all the populations were as follows: *G* vs. *A*: OR =1.16, 95% CI =1.08-1.25, *P*<0.0001; *GG* vs. *AA*: OR =1.29, 95% CI =1.12-1.50, *P*=0.0006; *GA* vs. *AA*: OR =1.19, 95% CI =1.07-1.32, *P*=0.001; *GG* + *GA* vs. *AA*: OR =1.24, 95% CI=1.11-1.39, *P*=0.0001 and *GG* vs. *GA*+*AA*: OR =1.15, 95% CI=1.02-1.30, *P*=0.02. The main results of overall analyses were shown in Table 3. 17 studies were conducted on Caucasians, 14 on Asians, 2 on Africans and 1 on Latin Americans. Subsequently, stratified analysis by ethnicity was conducted to get more clarifications. In the subgroup analysis, a significantly increased risk of RA was observed among the Asian population in all genetic comparisons except heterozygote comparison (*G* vs. *A*: OR =1.27, 95% CI =1.10-1.47, *P*=0.001; *GG* vs. *AA*: OR =1.58, 95% CI =1.24-2.01, *P*=0.0002; *GG* + *GA* vs. *AA*: OR =1.33, 95% CI=1.17-1.51, *P*<0.0001; *GG* vs. *GA*+*AA*: OR = 1.15, 95% CI =1.02-1.30, *P*=0.02). In Latin American population, rs231775 SNP was a significant risk factor of RA, but it only included single study and the result might be incredible. Besides, no association of the rs231775 SNP with RA risk was found among the Caucasian population in all genetic comparisons when the Elshazli’s study [24] was excluded because of its heterogeneity (*G* vs. *A*: OR =1.07, 95%CI =0.99-1.15, *P* =0.08; *GG* vs. *AA*: OR = 1.07, 95% CI = 0.92–1.23, *P*=0.37; *GA* vs. *AA*: OR = 1.15, 95% CI =1.00-1.31, *P*=0.05; *GG* + *GA* vs. *AA*: OR =1.14, 95% CI=1.00-1.29, *P*=0.05 and *GG* vs. *GA*+*AA*: OR =1.00, 95% CI=0.90–1.11, *P*=0.98) (Table 3 and Figure 3). There was no remarkable association between rs231775 SNP and RA in Africans. The results were summarized in Table 3 and Figure 3. These data with moderate heterogeneity employed the random-effect model for the synthesis.

### Meta-analysis of CTLA-4 318C/T (rs5742909) SNP and RA susceptibility

**Figure 4:**
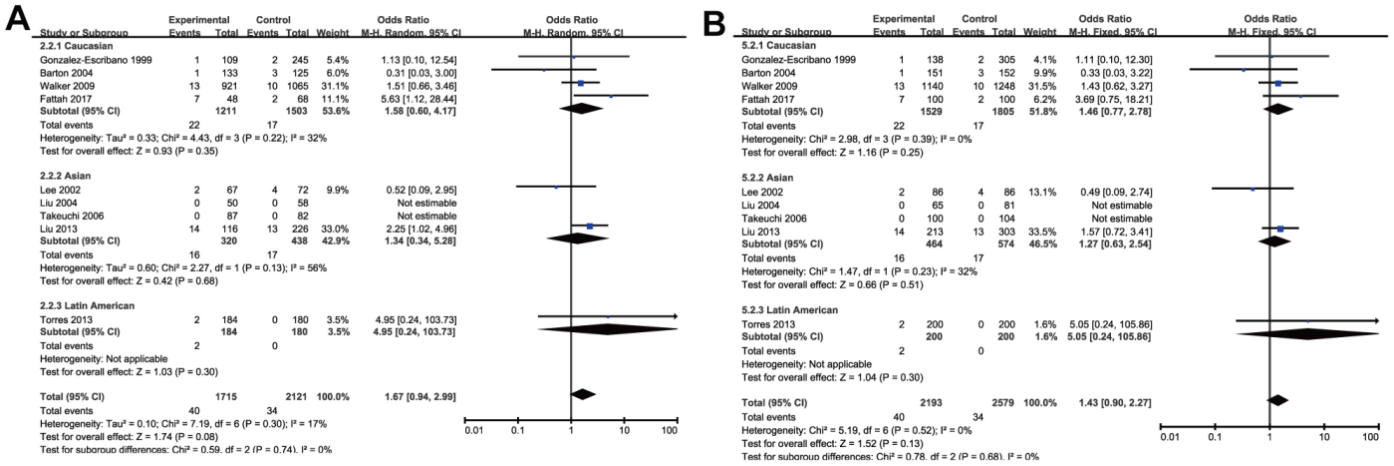
Forest plot of the association between rs574299 polymorphism and RA risk under the homozygous (**A**) and recessive model (**B**).

Through the pooled analysis of genetic data of 2477 patients and 2941 controls in a total of 10 studies, of which 5 were conduct on Caucasians, 4 on Asians, and 1 on Latin Americans, no significant associations between rs5742909 SNP and RA in the overall pooled results were found among all populations for the allelic and genotypic comparisons (*T* vs. *C*: OR =1.21, 95% CI =0.93-1.57, *P*=0.15; *TT* vs. *CC*: OR =1.71, 95% CI =1.08-2.73, *P*=0.08; *TC* vs. *CC*: OR =1.19, 95% CI =0.84-1.69, *P*=0.33; *TT*+*TC* vs. *CC*: OR =1.19, 95% CI=0.84-1.69, *P*=0.33 and *TT* vs. *TC*+*CC*: OR =1.43, 95% CI=0.90-2.27, *P*=0.13) (Table 3 and Figure 4). Meanwhile, the subgroup analysis by ethnicity did not indicate any remarkable associations in all genetic models (Table 3). As the heterogeneity of genetic model existed, random effect model in this part was used to make a reliable result.

### Heterogeneity analysis and publication bias

**Figure 5:**
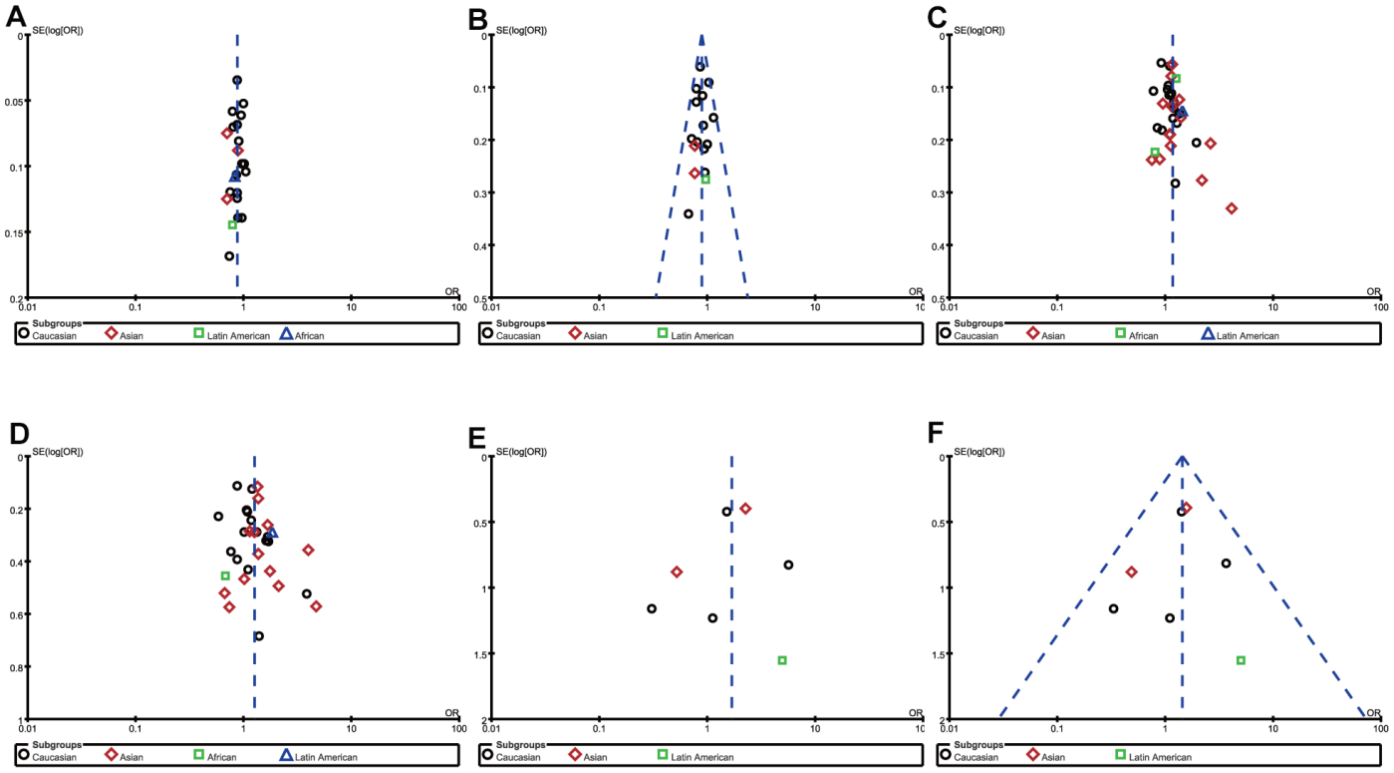
Funnel plot of the association between RA risk and rs308724 polymorphism under the allelic (**A**) and recessive model (**B**), rs231775 polymorphism under the allelic (**C**) and homozygous model (**D**), and rs574299 polymorphism under the homozygous (**E**) and recessive model (**F**).

To ensure the reliability of the results, we first evaluate the heterogeneity (by *I^2^*) and found that heterogeneity existed in some genetic models of rs231775 SNP and rs5742909 SNP (Table 3). In order to minimize heterogeneity, the following methods were carried out in this meta-analysis. On the one hand, the random-effect models were exploited in the genetic models with moderate heterogeneity(*I^2^*>50%). On the other hand, sensitivity analysis was adopted to evaluate the effect of a single study on the pooled ORs by removing each study in turn from the pooled analysis. Although the heterogeneity had not changed obviously, the P values for pooled ORs under allelic comparison, heterozygous comparison and dominant comparison were reversed when the study [24] led by Elshazli R was removed. Therefore, we deleted this study and recalculated the relevant ORs and 95%CIs to harvest a stable and credible outcome (Figure 3). The funnel plots were used to investigate publication bias and the outlines of the funnel plots appear to be symmetrical (Figure 5). For rs231775 SNP, the asymmetry of the funnel plot was attributed to Zhou et al.’s study [45] which was published in Chinese. HWE estimation indicated that allele or genotype frequencies were deviant from HWE in control group in the Liu et al.’s, Gonzalez-Escribano et al.’s and Sameem et al.’s studies [25, 29, 38], but the results of synthesis analysis were not substantially inversed. Hence, we didn't remove these studies from the meta-analysis.

## DISCUSSION

To our knowledge, this was the first meta-analysis to investigate the association between the three most-often SNPs of *CTLA-4* and RA susceptibility. From the data integration of 66 studies in 21681 cases and 23457 controls, we found that the rs3087243 SNP decreased the risk of RA risk in Caucasians and Asians, the rs231775 SNP of *CTLA-4* increased RA risk in Asians but not in Caucasians and Africans, and the rs5742909 SNP was not significantly associated with RA risk in both Caucasians and Africans.

The *CTLA-4* gene, located on chromosome 2q33, encodes a 223 amino acid receptor protein on T cell surface which is responsible for T cell immune regulation. As an antagonist of the costimulatory receptor CD28 which binds the same ligand B7 as CTLA-4, CTLA-4 with higher affinity transmits an inhibitory signal and subsequently plays a suppressive role in regulating T-cell activation [57], which suggests it is involved in the pathological processes of many autoimmune disorders [12–15]. It is widely believed that RA is a T cell-mediated autoimmune disease [58], of which the chronic inflammation and damage of the joints are typical [1]. Although a great many genes whose protein products are critical to T cell function don’t have genetic associations with RA, the effect of *CTLA-4* on RA pathogenesis has attracted growing attentions.

Previous research had found that serum levels of soluble CTLA-4 were increased in RA patients and had a positive correlation with Disease Activity Score in RA patients and even proposed that serum levels of CTLA-4 could serve as a new marker of RA disease activity [59, 60]. Besides, function experiments *in vivo* indicated that gene delivery of CTLA4 by intra-articular injection could alleviate experimental arthritis [61]. Furthermore, CTLA-4Ig administration on RA synovial macrophages and T helper cells downregulated the production of proinflammatory cytokines, and these evidences suggested that CTLA-4 could be a treatment target for RA [62, 63]. In fact, blockade of CTLA-4 by CTLA-4Ig had been successfully applied to treatment for RA [64].

As we all know, the protein level, structure and function are determined in large part by gene. Apart from these function research, numerous studies on correlation between *CTLA-4* and RA risk from gene level also had been conducted to investigate genetic factors [8, 9, 13–15, 17–20, 24–56]. However, the results were inconsistent or contrary likely due to the various ethnic background, disparate geographic environment, limited sample size, insufficient data and so on. Thus, it was urgently necessary to perform a comprehensive up-to-date meta-analysis as an effective methodology to draw an overall objective appraisal on the association between *CTLA-4* polymorphism and RA susceptibility.

In the present meta-analysis, we extracted 66 studies with 21681 cases and 23457 controls to inspect the correlation between three most-often SNPs in the *CTLA-4* gene and the risk of RA. There were 22 studies with 16394 cases and 17453 controls for rs3087243 SNP, 34 studies with 11452 cases and 12444 controls for rs231775 SNP, and 10 studies with 2477 cases and 2941 controls for rs5742909 SNP. For rs3087243 polymorphism, our findings demonstrated a decreased susceptibility of RA both in total and in Caucasians in any gene mode. In total, carriers with allele *A* reduced an approximate 13% risk of RA than ones with allele *G* and genotype *AA* reduced 20% or so than genotype *GG*. Moreover, a decreased susceptibility of RA was respectively also found among Asians in the allele and homozygote comparison and among Latin Americans in the heterozygote and dominant comparison. However, only one study was included in Latin Americans and Asians so it needed to enlarge sample size to further research. For rs231775 polymorphism, significant association did exist among the whole population in all genetic models except recessive model: compared with allele *A* and genotype *AA*, allele *G* and genotype *GG* and *GA* respectively was associated with an increased risk of RA. The same association was observed in Asians and Latin Americans in the subgroup analysis. On the contrary, no significant association between rs231775 SNP and RA risk could be detected in Caucasians and Africans using any gene model after excluding the Elshazli R’s study [24] with the apparent heterogeneity. Here, it should be noted that only one or two case–control study was included in Africans and Latin Americans, so the conclusions were not particularly convincing. For rs5742909 polymorphism, no significant association between this locus polymorphism and RA risk was observed among any population in any model. Although the heterogeneity existed in some genetic model, but no obvious change had happened in heterogeneity and *P* value for the pooled ORs when each study was individually removed by sensitivity analysis.

With regard to the diverse results of the same SNP on different populations, it might be attributed to clinical and genetic real heterogeneity of RA, interaction of genetic background and region environment, and even lack of vigorous statistical power. Besides, it was noteworthy that one important factor for the diverse and disparate results was linkage disequilibrium (LD). These *CTLA-4* SNPs might be not definitely the causative alleles, but they were likely to be in LD with the causative alleles which were yet unidentified. And, LD was different between ethnic and racial groups.

It should be pointed out that previous several meta-analyses have summarized the effect of *CTLA-4* polymorphism on RA risk [21–23, 65]. But a few points need to be taken notice. On one hand, the previous conclusions were discordant as the following: the conclusion of Li’s (2014) study [65] on the association of rs231775 SNP of *CTLA-4* with RA was contrary to the others; the genetic models which indicated significant association were diverse in these analyses. These differences were mainly originated from divergent diagnostic criteria, limited number of studies and sample sizes. On the other hand, all these meta-analyses focused on only one of the three well-studied loci except Li’s study [23] on two. As we all know, the expression and function of the protein are determined by the whole gene. Therefore, it is of great necessity to investigate simultaneously the effect of all the 3 SNPs on RA risk to obtain an overall evaluation. Besides, the number of included studies in previous meta-analyses was small. Some original association studies [9, 15, 24–27] have emerged in the past few years and they can be incorporated. Taking these points into considerations, we updated the meta-analysis to achieve a more valid and comprehensive estimation on the association of *CTLA-4* gene and RA susceptibility.

However, some limitations of our study should be acknowledged. Firstly, the small sample size in some studies and the limited studies for some stratified analysis were not sufficient enough to detect the relationship. Especially, the results of populations including only one study should be interpreted with caution. Secondly, we only investigated the role of three loci polymorphisms. As *CTLA-4* gene had various SNPs, the function of protein CTLA-4 depended on the whole gene and RA was a multigene susceptibility disease, more SNPs of *CTLA-4* should be included. Thirdly, certain degree of heterogeneity still existed in rs5742909 polymorphism and some genetic models. Although the elimination of each single study did not distinctly alter the *P* value, the results must still be treated cautiously. Fourthly, inadequate raw data in some studies result in the inability to calculate the number of the genotypes and perform stratified analysis by age, gender and autoantibody status such as RF etc. As a consequence, any potential gene-environment and gene-gene interactions could not be accessed.

In conclusion, this meta-analysis suggested that rs3087243 polymorphisms were corelated with a reduced RA risk in both Asian and Caucasian populations, rs231775 polymorphisms was associated with an increased risk of RA in Asians, and rs5742909 polymorphism had no significant association with RA risk. Larger-scale studies of populations with different ethnicities are encouraged to validate the role *CTLA-4* played in the pathogenesis of RA.

## MATERIALS AND METHODS

This meta-analysis was performed according to the Preferred Reporting Items for Systematic Reviews and Meta-Analyses (PRISMA) guidelines [66].

### Search strategy

From the databases PubMed, EMBASE, Web of Science and, the China National Knowledge Infrastructure (CNKI) and Wan Fang data, a comprehensive systematic literature retrieval was conducted to derive all relevant studies published before 10 October, 2020 (the search was constantly updated to submission). The following terms as Medical Subject Heading and free words were applied: “*CTLA-4* or cytotoxic T lymphocyte antigen-4” and “single nucleotide polymorphism or polymorphism or variant or variation” and “rheumatoid arthritis or RA”. The bibliographic lists of included studies were also browsed for potential related studies. There were no restrictions on language and publication date in this study.

### Inclusion and exclusion criteria

The current meta-analysis used the following inclusion criteria to screen available literatures: 1) case-control study; 2) evaluation of the associations between *CTLA-4* (rs3087243, rs231775 and rs5742909)polymorphism and RA risk; 3) with sufficient data for extract odds ratios (ORs) and 95% confidence intervals(CIs); (4) with reported allele or genotype numbers or frequencies in cases and control group; 5) with a clear diagnostic criteria. Accordingly, we excluded meaninglessness literatures if they had the following trait: 1) case report, comment, animal studies and conference abstracts; 2) with no detailed allele or genotype data; 3) duplications or no controls.

### Data extraction and assess of quality

Two independent investigators respectively conducted a literature search according to the above search strategy, screened each article based on the predesigned inclusion and exclusion criteria, and extracted data from these eligible studies. It would be settled by discussion with the third party when the disagreement between investigators occurred. The following information was collected from every paper: 1) first author's surname, 2) the year of publication, 3) country or region of origin, 4) ethnicity, 5) total numbers of cases and controls, 6) genotype method, 7) diagnostic criteria, 8) polymorphism locus, 9) allele distribution or/and genotype distribution.

The methodological quality of included studies was accessed in light of the Newcastle–Ottawa Scale (NOS) for the evaluation of observational studies [67]. In brief, three broad perspectives were evaluated using the Star system (http://www.ohri.ca/programs/clinical_epidemiology/oxford.asp). Any divergence between two investigators was solved by discussion until agreement was reached.

### Statistical analysis

The strength of association of rs231775, rs5742909 and rs3087243 SNPs with RA risk was appraised via estimating ORs with their corresponding 95% CIs. For each SNP, the pooled ORs were calculated individually for five gene models (allele model, homozygote model, heterozygote model, dominant model and recessive model). The Z test was used to evaluate the significance of the pooled ORs. p<0.05 was judged as statistically significant difference. Statistical Heterogeneity between studies was assessed by Chi square and *I*^2^ values which range from 0% to 100%. 25%, 50%, and 75% were regarded as respectively low, moderate, and high level [68, 69]. The random -effect model was employed when the value of *I*^2^ was more than 50%. If not, the fixed effect model was employed. Hardy–Weinberg equilibrium (HWE) was tested in the control group for all studies by Chi-square test to judge whether the selection bias existed. Potential publication bias was examined by funnel plots. Besides, the current meta-analysis had carried out subgroup analyses by the racial descent to assess the effects of ethnic background.

The above statistical analyses were performed using Review Manager 5.3 software (Nordic Cochrane Centre, Cochrane Collaboration, Copenhagen). All the P values were 2-sided and P<0.05 signified statistically significance.

## Supplementary Material

Supplementary Table 1
