# Supplementary material for: Association between CTLA-4 gene polymorphism and risk of rheumatoid arthritis: a meta-analysis
Source: Aging (Albany NY). 2021 Aug 2;13(15):19397–414. doi: 10.18632/aging.203349 (PMC8386564; doi:10.18632/aging.203349)
Supplement: Supplementary Table 1 [file aging-13-203349-s001.pdf]

SUPPLEMENTARY TABLE

Supplementary Table 1. Quality assessment of included studies according to the Newcastle-Ottawa Scale.

Study	Selection				Comparability	Exposure		
	Adequate definition of cases	Representativeness of cases	Selection of controls	Definition of controls	Control for important or additional factor	Exposure assessment	Same method of ascertainment for all subjects	Non-response rate
Seid C	*	-	*	*	*	*	*	*
Gonzalez-Escribano MF	*	-	-	*	*	*	*	*
Matsushita M	*	-	*	*	*	*	*	*
Barton A 2000	*	-	*	*	*	*	*	*
Yanagawa T	*	-	-	*	*	*	*	*
Hadj KH	*	-	*	*	*	*	*	*
Milicic A	*	-	*	*	**	*	*	*
Lee YH	*	-	-	*	*	*	*	*
Vaidya B	*	-	-	*	*	*	*	*
Lee CS	*	-	-	*	*	*	*	*
Barton A 2004	*	-	*	*	*	*	*	*
Liu MF	*	-	-	*	*	*	*	*
Miterski B	*	-	*	*	*	*	*	*
Orozco G	*	-	*	*	*	*	*	*
Feng ZL	*	-	-	*	*	*	*	*
Lei C	*	-	*	*	**	*	*	*
Plenge RM	*	-	*	*	**	*	*	*
Zhernakova A	*	-	-	*	*	*	*	*
Suppiah V	*	-	*	*	*	*	*	*
Takeuchi F	*	-	-	*	*	*	*	*
Zhou Y	*	-	-	*	*	*	*	*
Costenbader KH	*	-	*	*	*	*	*	*
Tsukahara S	*	-	*	*	**	*	*	*
Barton A 2009	*	-	*	*	**	*	*	*
Daha NA	*	-	-	*	**	*	*	*
Kelley JM	*	-	*	*	*	*	*	*
Walker EJ	*	-	*	*	**	*	*	*
Muñoz-Valle JF	*	-	-	*	*	*	*	*
Plant D	*	-	*	*	**	*	*	*
Benhatchi K	*	-	-	*	*	*	*	*
Danoy P	*	-	*	*	*	*	*	*
El-Gabalawy	*	-	*	*	*	*	*	*
AIFadhli S	*	-	-	*	*	*	*	*
Liu CP	*	-	*	*	*	*	*	*
Tang MJ	*	-	*	*	**	*	*	*
Torres-Carrillo N	*	-	*	*	*	*	*	*
Sameem M	*	-	-	*	*	*	*	*
Elshazli R	*	-	-	*	*	*	*	*
Luterek-Puszyńska K	*	-	*	*	**	*	*	*
Vernerova L	*	-	*	*	**	*	*	*
Fattah SA	*	-	*	*	*	*	*	*
Schulz S	*	-	-	*	*	*	*	*

A study could be awarded a one or zero star for every item except for the item “Control for important factor or additional factor”.
